# Exploring the patient experience of locally advanced or metastatic pancreatic cancer to inform patient-reported outcomes assessment

**DOI:** 10.1007/s11136-019-02233-6

**Published:** 2019-07-04

**Authors:** Joseph M. Herman, Helen Kitchen, Arnold Degboe, Natalie V. J. Aldhouse, Andrew Trigg, Mary Hodgin, Amol Narang, Colin D. Johnson

**Affiliations:** 1 Department of Radiation Oncology and Molecular Radiation Sciences, Johns Hopkins University, Baltimore, MD USA; 2 DRG Abacus, Clinical Outcomes Assessment, Manchester, UK; 3 AstraZeneca, Gaithersburg, MD USA; 4 Adelphi Values, Macclesfield, UK; 5 Department of Surgery, Advanced Practice Nursing, Johns Hopkins University, Baltimore, MD USA; 6 Surgical Unit, University of Southampton, Southampton, SO16 6YD UK

**Keywords:** Pancreatic cancer, Qualitative research, Oncology, Patient experience, Patient-reported outcome (PRO), Disease conceptual model

## Abstract

**Purpose:**

Pancreatic cancer and its treatments impact patients’ symptoms, functioning, and quality of life. Content-valid patient-reported outcome (PRO) instruments are required to assess outcomes in clinical trials. This study aimed to: (a) conceptualise the patient experience of pancreatic cancer; (b) identify relevant PRO instruments; (c) review the content validity of mapped instruments to guide PRO measurement in clinical trials.

**Methods:**

Qualitative literature and interviews with clinicians and patients were analysed thematically to develop a conceptual model of patient experience. PRO instruments were reviewed against the conceptual model to identify gaps in measurement. Cognitive debriefing explored PRO conceptual relevance and patients’ understanding.

**Results:**

Patients in the USA (*N *= 24, aged 35–84) and six clinicians (from US and Europe) were interviewed. Pre-diagnosis, pain was the most frequently reported symptom (*N *= 21). Treatments included surgery, radiation, chemotherapy, and immunotherapy. Surgery was associated with acute pain and gastrointestinal symptoms. Chemotherapy/chemoradiation side effects were cyclical and included fatigue/tiredness (*N *= 21), appetite loss (*N *= 15), bowel problems (*N *= 15), and nausea/vomiting (*N *= 15). Patients’ functioning and well-being were impaired. The literature review identified 49 PRO measures; the EORTC QLQ-C30/PAN26 were used most frequently and mapped with interview concepts. Patients found the EORTC QLQ-C30/PAN26 to be understandable and relevant; neuropathic side effects were suggested additions.

**Conclusions:**

This is the first study to develop a conceptual model of patients’ experience of metastatic/recurrent pancreatic cancer and explore the content validity of the EORTC QLQ-C30/PAN26 following therapeutic advances. The EORTC QLQ-C30/PAN26 appears conceptually relevant; additional items to assess neuropathic side effects are recommended. A recall period should be stated throughout to standardise responses.

**Electronic supplementary material:**

The online version of this article (10.1007/s11136-019-02233-6) contains supplementary material, which is available to authorized users.

## Background

Pancreatic cancer is the fourth or fifth highest cause of cancer-related deaths in most developed countries [1]. At least 80% of patients diagnosed with pancreatic cancer will have locally advanced or metastatic disease, and receive palliative and definitive therapies designed to reduce symptoms, such as back/abdominal pain, jaundice, lack of energy and weight loss [2]. Pancreatic cancer and its treatment have a negative impact on patients’ functioning, well-being and other aspects of health related quality of life (HRQoL) [3, 4].

Patient-reported outcome (PRO) instruments are increasingly important for assessing outcomes in pancreatic cancer care and research [5] directly from the patient perspective and provide important information to evaluate outcomes in clinical trials [6]. However, PRO instruments vary significantly with respect to their development and validation and there are many factors to consider when selecting a PRO instrument including target population characteristics (e.g. tumour location, disease stage), treatment, timing of assessment, clinical setting, study purpose, the research question, and how well the PRO instrument assesses symptoms and impacts that are meaningful to patients. To minimise measurement error, the content validity and psychometric properties of a PRO instrument should be well understood within the population of interest and context of use [7–9].

To evaluate content validity, it is important to understand whether the PRO instrument measures concepts that are important to patients [10].

One method to evaluate this is to perform qualitative exploration with patients. However, there is a paucity of published qualitative studies reporting the lived experience of patients with pancreatic cancer and even fewer studies that report the experience of patients with metastatic/recurrent pancreatic cancer.

This study therefore sought to explore and understand the experience of patients living with metastatic/recurrent pancreatic cancer, to develop a conceptual model to reflect patients’ experience, and to evaluate the content validity of selected PRO instruments for assessing patient outcomes.

## Methods

This study comprised a qualitative literature review and qualitative interviews with clinicians and patients to develop a disease conceptual model. The PRO instruments most frequently used in pancreatic cancer studies were identified via an additional literature review. The conceptual relevance of the PRO instruments was explored by comparing and contrasting the qualitative findings with the conceptual model. The content validity of the PRO instruments was further explored though cognitive debriefing interviews.

### Qualitative literature review

A search was conducted of the Medline, EMBASE, and PsycINFO databases and conference proceedings from the International Society for Pharmacoeconomics and Outcomes Research (ISPOR) and International Society of Quality of Life Research (ISOQOL), to identify published qualitative research describing the patient experience of pancreatic cancer (at any stage of illness).

Electronic literature searches were conducted in July 2014 using pre-defined search terms, e.g. pancreatic cancer AND qualitative interview (full search terms are presented in the Supplementary Information). Articles meeting eligibility that were published in English between 2000 and 2014 were retained for full review to identify recent literature and detect the experiences of patients treated with modern chemotherapy regiments. Due to an expected paucity of formal published qualitative literature, two social media websites (http://community.macmillan.org.uk and http://www.cancercompass.com) deemed to be reputable sources with relevant content, audience and level of activity, were identified through a targeted, non-systematic search and selected as supplementary sources of information. Social media posts were subject to an insights-driven search and review to identify quotes related to lived experience. Quotes from the literature and social media review were thematically analysed to identify concepts and themes.

### PRO instrument search

The PubMed, PROQOLID and PROLabels databases were searched on 10th September 2014 to identify PRO instruments used in pancreatic cancer studies using search terms, e.g. ‘patient-reported outcome’ AND ‘pancreatic cancer’) (see Appendices in Supplementary Information). The PRO instruments with highest frequency of use in pancreatic cancer studies were identified for including in cognitive debriefing interviews.

### Qualitative clinician interviews

Semi-structured telephone interviews were conducted with clinicians who were experienced in treating pancreatic cancer. A semi-structured interview guide was developed with a particular focus on diagnosis and treatment. Interviews lasted approximately 60 min and were conducted by an experienced qualitative interviewer in December 2014. As the interviews were exploratory to inform the patient interview guide no independent ethics review was sought. However, the standards of Good Clinical Practice were followed; potential participants were provided with written information about the study, and written informed consent was obtained, and data obtained were confidential and anonymised.

### Qualitative patient interviews

A study protocol was reviewed and approved by Johns Hopkins Institutional Review Board (IRB) (13-Nov-2015, amendment 29-Feb-2016 ref: J-15139). Clinicians at Johns Hopkins University Hospital recruited eligible patients (Table 1). Written informed consent was obtained and an honorarium was provided to patients.

**Table 1:** Patient interview inclusion and exclusion criteria

Criteria	Inclusion	Exclusion
Demographics	• Male or female of any race and at least 18 years of age on the day of the research interview	• < 18 years
Diagnosis	• Patient has a confirmed diagnosis of locally advanced or metastatic pancreatic cancer (TNM Stage 4) • Patient has received treatment in the past 12 months for their locally advanced or metastatic pancreatic cancer	• Patient has had another malignancy within 5 years except for non-invasive malignancies such as cervical carcinoma in situ, non-melanomatous carcinoma of the skin or ductal carcinoma in situ of the breast that has/have been surgically cured
Concomitant illnesses	• N/A	• Patient has untreated or symptomatic CNS metastases • Patient has an active infection including hepatitis B, hepatitis C, or HIV
Physical and psychological wellness	• Participant has a WHO Performance Status of 0 or 1 • In the opinion of the patient’s clinician, patient has the cognitive, reading and linguistic capacities sufficient to allow her/him to actively participate in a 90 min interview conducted in US-English	• Patient has a significant psychiatric or physical co-morbid condition that would, in the opinion of the patient’s clinician, prevent the patient’s participation in this study • Patient is engaged in or has prior documented history of active substance abuse in the last 12 months
Informed consent	• Patient has personally read, signed and dated a legally effective written informed consent form prior to admission to the study, in addition to any locally required authorization	• Patient is unwilling or unable to comply with the requirements of the study

*CNS* central nervous system, *HIV* human immunodeficiency virus, *N/A* not applicable, *WHO* World Health Organization

### Interview procedure

A semi-structured interview guide was developed by experienced qualitative researchers and clinicians, informed by data gathered from the qualitative literature review, social media review and clinician interviews. The guide comprised concept elicitation and cognitive debriefing sections, utilising standard methodology [11] in line with the FDA PRO Guidance [9].

During concept elicitation, patients were asked open-ended questions about their pancreatic cancer journey, from diagnosis to treatment, and its impact on their daily lives.

During cognitive debriefing, patients completed a paper and pen version of the PROs using a ‘think aloud’ technique [11], to explore their understanding of each instruction, item, response scale/option and recall period. Patients were also asked if any important experiences were missing from the PRO.

In recognition of the debilitating effects of metastatic pancreatic cancer and its treatment, patients could complete the interview in two separate sessions if preferred. Interviews lasted for approximately 90 min and were conducted between December 2015 and November 2016.

## Analysis

Interviews were audio-recorded and transcribed verbatim. Concept elicitation data were analysed thematically [12], facilitated by ATLAS ti.v7. Analysts assigned descriptive codes to sections of text pertaining to common themes. Codes were applied iteratively as new concepts and themes emerged. Data were collated with the qualitative literature review/social media findings and clinician interview data to further refine the conceptual model.

Cognitive debriefing data were analysed using framework coding to assess conceptual relevance, item interpretation, and appropriateness of instructions, response scales/options, and recall period. To evaluate conceptual coverage, items in the PRO were compared and contrasted with the conceptual model.

### Conceptual saturation

Determination of an adequate sample size was explored through the principle of conceptual saturation [13]. Published evidence suggests that a sample of 12–15 is usually the minimum sufficient [13, 14] and including a sample size of more than 25 is not deemed beneficial [15].

To explore saturation, transcripts were divided into three sets of eight based on the chronological order of interview completion. The first mention of each identified concept was reviewed. If no new concepts were identified in the third set of interviews, saturation was deemed to have been attained.

## Results

### Qualitative literature review

A total of 5,899 articles were identified and 5886 excluded at first pass following a title/abstract review. Thirteen articles met eligibility criteria for full text review, of which *N *= 6 articles were retained for data extraction. A PRISMA flow diagram presented in the supplementary Information. From the two social media websites *N *= 50 relevant blog entries (by patients with pancreatic cancer (*N *= 42) or their caregivers (*N *= 8) were extracted for further review.

### Qualitative clinician interviews

Six clinicians from the USA (*N *= 3) and the EU (*N *= 3) participated; their current roles included medical oncologist (*N *= 4), radiation oncologist (*N* = 1) and surgeon (*N *= 1). All clinicians worked in university hospitals (*N *= 5) or academic cancer centres (*N *= 1) and had been involved in diagnosis, treatment and research for between 11 and 30 years.

### Qualitative patient interviews

A total of 24 patients were interviewed; sample demographic and clinical characteristics are summarised in Tables 2 and 3.

**Table 2:** Demographic characteristics of patients participating in patient interviews

Demographic characteristic	Participants, *N* (%) (*N *= 24)
**Gender** ^a^	
Male	9 (38)
Female	15 (63)
**Age, years** ^a^	
Mean (SD) [range]	62 (10) [35–84]
**Ethnicity**	
Black or African American	4 (17)
Caucasian or white	20 (83)
**Daily living support received from informal caregiver?** ^a^	
Yes	23 (96)
No	1 (4)
**Time with informal caregiver, hours/week**	
> 15	22 (92)
< 15	1 (4)
Not applicable	1 (4)
**Education**	
Bachelor/graduate degree or higher	16 (67)
High school diploma or equivalent	6 (25)
Some graduate work	1 (4)
Other	1 (4)
**Work status**	
Employed	11 (46)
Full time homemaker	2 (8)
Not working due to pancreatic cancer	3 (13)
Retired	7 (29)
Not working, reason unclear	1 (4)

^a^Percentages do not total 100% due to rounding

**Table 3:** Clinical characteristics of patients participating in patient interviews

Clinical characteristic	Participants, *N* (%) (*N *= 24)
**Tumour location**	
Head	18 (75)
Body and tail	3 (13)
Body	1 (4)
Body and head	1 (4)
Head and neck	1 (4)
**Primary tumour stage**	
T3	14 (58)
T4	10 (42)
**Regional lymph node stage** ^a^	
N0	6 (25)
N1	9 (38)
NX	9 (38)
**Status of pancreatic tumour**	
Locally advanced	18 (75)
Metastatic	5 (21)
Tumour recurrence/new lesion	1 (4)
**Experienced tumour progression**	
Yes	11 (46)
No	11 (46)
Undetermined	2 (8)
**Time since diagnosis**	
≤ 2 months	2 (8)
3–6 months	7 (29)
7–12 months	7 (29)
1–2 years	4 (17)
2 years	4 (17)
**Type of current treatment**	
Chemotherapy	15 (63)
Immunotherapy	1 (4)
Not currently receiving treatment	8 (33)
**Treatment received previously or currently**	
Chemotherapy	24 (100)
Of which:	
FFX	20 (83)
Gemcitabine + Abraxane	7 (29)
Gemcitabine	4 (17)
5FU + Leucovorin	1 (4)
Gemcitabine + Bevacizumab	1 (4)
GTX	1 (4)
Radiotherapy	9 (38)
Of which:	
Chemoradiotherapy	4 (17)
Surgery (Whipple)	6 (25)
**Comorbidities** ^b^	
High blood pressure	13 (54)
Diabetes	11 (46)
Heart disease	1 (4)

*5FU* fluorouracil, *FFX* folfirinox, *GTX* gemcitabine, docetaxel and capecitabine

^a^Percentages do not total 100% due to rounding

^b^Categories are not mutually exclusive; percentages do not total 100%

#### Conceptual saturation

Analyses identified 57 concepts, of which 50 arose spontaneously in the first two sets of patient interviews (Supplementary Information). Two concepts arose in the clinician interviews only. Five new concepts arose in the third set of patient interviews but were mentioned by ≤ 2 patients and seemed to be specific to those patients’ circumstances. In-depth, rich data were obtained which provided a comprehensive insight into pancreatic cancer experience. It was therefore concluded that saturation was met after *N *= 24 interviews.

### Patient experience of pancreatic cancer

**Fig. 1:**
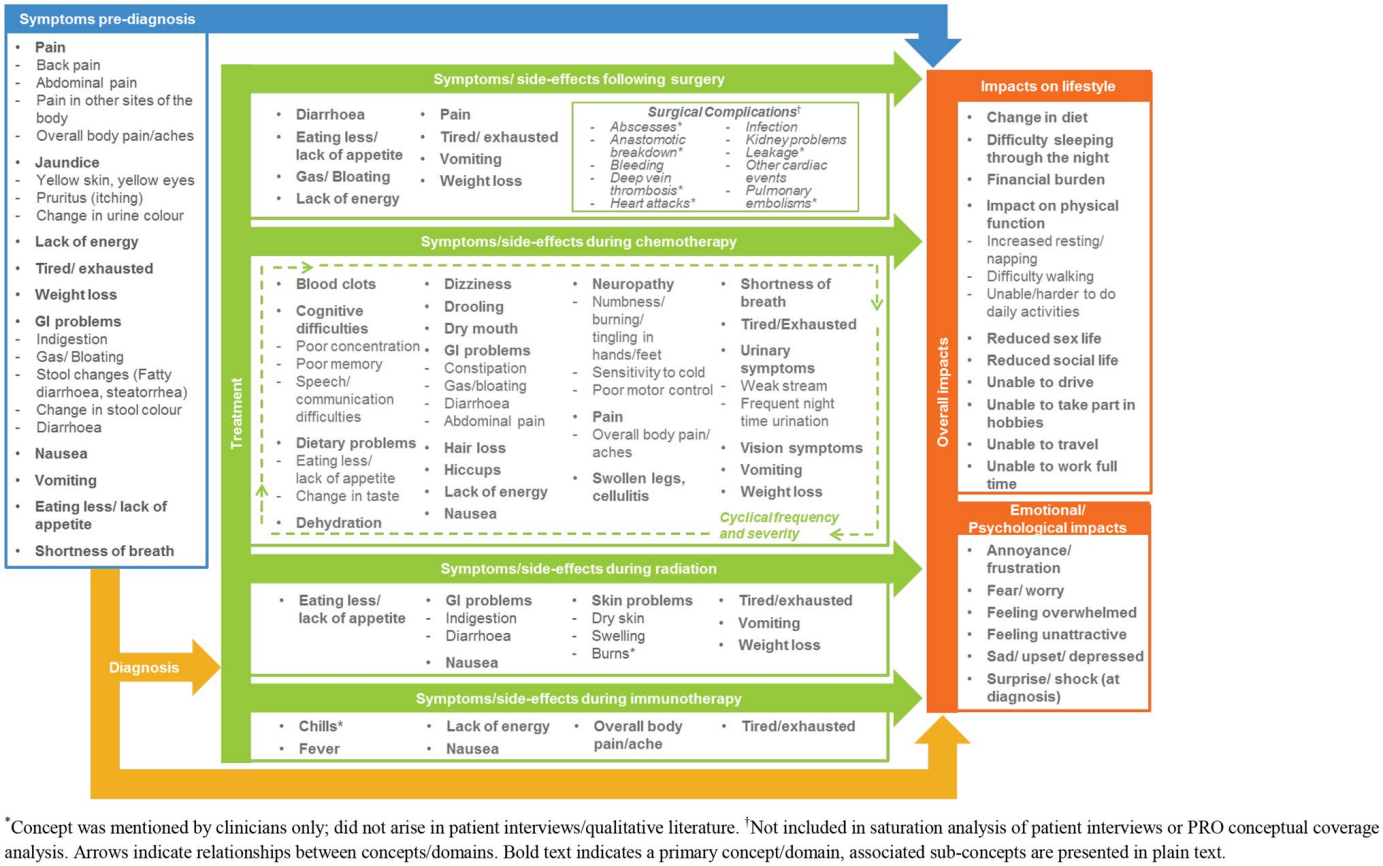
Conceptual model. *Concept was mentioned by clinicians only; did not arise in patient interviews/qualitative literature. ^†^Not included in saturation analysis of patient interviews or PRO conceptual coverage analysis. Arrows indicate relationships between concepts/domains. Bold text indicates a primary concept/domain, associated sub-concepts are presented in plain text

A conceptual model was developed based upon the qualitative literature review and concept elicitation data from patient and clinician interviews (Fig. 1). Patients’ experience of pancreatic cancer was represented by pre-diagnosis symptoms, symptoms/side effects experienced during treatment, and the lifestyle and emotional/psychological impacts resulting from diagnosis and treatment.

### Pre-diagnosis

Pain was the most frequently reported symptom during discussion of pre-diagnosis experiences, “*I had mid*-*thoracic back pain at night that just wouldn’t go away”* (01-15). Abdominal pain, back pain and upper gastrointestinal (GI) pain were the most commonly cited locations. Other commonly reported signs/symptoms included yellow skin/eyes, pruritus and urine colour change.

The literature review highlighted that some patients underestimated their symptoms or attributed them to other health problems [16]. Interviewed clinicians corroborated this and noted that patients may manage pain for months before they seek treatment (Clin-6-US).

### Diagnosis

Clinicians described diagnostic tests including surgical biopsy and/or scans to identify tumour location and disease stage. One clinician (Clin-1-EU) explained that the diagnostic tests he performed were dependent on presenting signs/symptoms. Diagnosis could be delayed for several weeks to several months due to the “*vague*” (Clin-3-US) and “*non*-*specific*” (Clin-3-US) nature of symptoms.

The initial impact of diagnosis for patients was emotional and psychological. Patients described feeling shocked, sad/depressed, and overwhelmed which sometimes resulted in denial, *“I was scared, I was upset, I said ‘this can’t happen to me, it happens to everybody else”* (01-08). Patients described feeling fearful and uncertain about the type and length of future treatment [17, 18]. Many patients felt worried about the future and the impact on their families. Following this, some described a process of acceptance during which they purposively developed an optimistic mind-set towards ‘fighting’ or ‘beating’ their cancer.

### Treatment

Surgery, chemotherapy, radiation and immunotherapy were described with each associated with potential symptoms/side effects. Clinicians made treatment recommendations based on the evidence for treatment efficacy in the context of patient performance status, disease stage and comorbidities.

#### Surgery

Clinicians described surgical treatment for patients with resectable pancreatic cancer and good performance status for whom surgery *“offers a small but real chance of cure.”* (Clin-3-US).

Clin-1-EU described patients’ main concerns prior to surgery; *“the anaesthetic, and who is going to do the operation. […] Just imagine you’re going to have a general anaesthetic and what’s going to worry you? […] Waking up.”*

Pain was the most immediate side effect of surgery reported by clinicians and patients. Patients also had experienced chronic or acute bowel or digestion problems because *“the plumbing is a little bit different”* (Clin-6-US), as well as reduced appetite and increased fatigue and weight loss, ‘*“It was quite painful, it took a lot out of me. I lost 25lbs in two and a half weeks. […], I was in good shape but it really pulled the weight off of me in a hurry. I was weak, […] I felt like I’d been hit by a bus”* (01-01). Clinicians noted that recovery to full strength may take 6-12 months, and the emotional, psychological and work impacts may be even longer lasting.

#### Chemotherapy

Clinicians described that almost all patients were treated with chemotherapy but the time of initiation and the type of chemotherapy drug administered was determined by stage and performance status. Common side effects reported by patients and clinicians included gastrointestinal problems, *“I would start with constipation and that would be for a day or two and it was bad. In the end I would switch over to having the gas and the bloating, [and] it would go over to diarrhoea and it would be very acidic diarrhoea so it was uncomfortable.” (01*-*04)*, nausea, cognitive difficulties, hair loss, and neuropathy, presenting as “*cold sensitivity or numb pins and needles in the hands and feet*” (Clin-3-US). Patients described side effects/symptoms that were cyclical in relation their treatment cycle. Clinicians confirmed that side effects were mostly ‘acute and transient’, lasting a few days after each treatment administration.

#### Radiation

Clinicians described radiotherapy as typically adjunctive to chemotherapy (‘chemoradiation’) used to reduce the risk of recurrence after surgery, to ‘downstage’ the disease ahead of surgical intervention, and to alleviate symptoms of late stage, metastatic disease. Common side effects reported by patients included tiredness/exhausted, “*I go for the radiation and I come home and go to bed for 28* *days [..] It took a lot out of me*.” (01-12) and lack of appetite, “*I didn’t want to eat anything at all, I forced myself to eat*.” (02-26).

One clinician reported that radiation skin damage may occur (Clin-4-US) but this was not reported by any interviewed patients.

#### Immunotherapy

Two clinicians discussed immunotherapy and biologic therapy and noted that there were no immunotherapy or biological drugs currently indicated for non-adjunctive treatment of pancreatic cancer. Clinicians noted that immunotherapy side effects may include lack of energy and tiredness/exhaustion. Only one patient interviewed had received immunotherapy and described a fever, body aches, and nausea, “*Afterwards I felt okay, […] I ended up feeling like I had the flu for two days so I was in bed for that […] I was nauseous but not nauseous to the point of throwing up*” (01-12).

### Overall impacts

The emotional/psychological impacts of diagnosis could prolong throughout treatment including fear/worry, feeling depressed/down. Patients also described the impact on their physical function, “*I was too weak to really go out and have a normal day, I had to be home and close to a couch where I could lie down because of fatigue.”* (01-09) and *‘before all this happened I would run foot races, 5* *k, 10* *k race, and I was no longer able to do that mainly because I didn’t have the energy to do it.”* (01-24). Patients reduced their social activities and some felt unable to make plans, ‘*A lot of times [my friends/family] say, “Do you want to go out so and so?” But it’s like, yes I’d love to go out to the lake, but I don’t know if I’m going to be able to stay out there, because I might have diarrhoea, […] sometimes it just comes out of nowhere. […] I don’t feel like I can make plans.”* (02-25). Many reduced or gave up working, ‘*I was working part time at [a] job that had required me to get up at 4.30 in the morning and be there by 5.30, and it was just too hard on my body, […] so I had to give it up’* (01-21).

### PRO review

The PubMed search identified 1030 abstracts which reported PRO use in pancreatic studies. From this and the PROQOLID and PROLabels searches, a total of 49 unique PRO instruments were identified that had been used in pancreatic cancer studies, of which 14 were utilised in ≥ two studies and/or were cancer-specific measures (listed in Supplementary Information). The EORTC QLQ-C30/PAN26 module and the FACT-Hep were identified as the two most commonly used PROs in pancreatic cancer studies.

The EORTC QLQ-C30 [19] is a 30-item questionnaire which measures global health, functioning and symptoms. A pancreatic-specific module, the PAN26 [20] included an additional 26 items which measure symptoms/side effects and functional and emotional issues specific to pancreatic cancer.

The FACT-Hep [21] is a 45-item questionnaire developed to assess physical, emotional, functional and social/family well-being in patients with hepatobiliary cancer.

Comparison with the conceptual model identified 26 concepts that were assessed by items in both the EORTC QLQ-C30/PAN26 and FACT-Hep. A total of 25 concepts were unique to the EORTC QLQ-C30/PAN26, and 15 concepts were unique to the FACT-Hep.

Some items unique to the EORTC QLQ-C30/PAN26 assessed proximal, functional or side effect/symptoms concepts (e.g. items 2 and 3, “*Do you have any trouble taking a short/long walk?* or item 15, “*Have you vomited?*”), whereas some items unique to the FACT-Hep could be considered to assess more distal impacts (e.g. item GS3, “I get support from my friends,” or item GF2, “my work (including work at home) is fulfilling”). Additionally, the EORTC QLQ-C30/PAN26 was specific for pancreatic cancer whereas the FACT-Hep was developed for hepatobiliary cancer. Therefore, the EORTC QLQ-C30/PAN26 was debriefed in patient interviews; findings are summarised in Table 4.

**Table 4:** Summary of EORTC QLQ-C30/PAN26 cognitive debriefing findings

Consideration	Findings
Conceptual relevance	• All items deemed relevant by patients • Most key symptom/side effect and impact concepts assessed, although some side effect assessments are missing (e.g. neuropathic symptoms)
Interpretation and understanding	• Instructions well understood Items generally well understood. Potentially problematic items included those assessing a ‘long’ or ‘short’ walk and ‘frequent bowel movements’
Response scale and options	• Mostly considered appropriate
Recall period	• Recall applied for Items 1–5 varied, due to absence of a specified recall period • The recall period of the remaining items was easily understood but not always adhered to throughout completion of questionnaire

### Conceptual relevance

All items of the EORTC QLQ-C30/PAN26 were deemed relevant by patients and most concepts measured had been identified within concept elicitation and published literature. Pain had been identified frequently and is assessed in six separate items. Other experiences that were important to patients were assessed including fatigue, gastrointestinal problems and dietary changes. Additionally, many emotional/psychological and lifestyle impacts were assessed by items in the EORTC QLQ-C30/PAN26.

However, some concepts identified in the interviews and included in the conceptual model were absent from the EORTC QLQ-C30/PAN26. No items were identified which assess neuropathic treatment-related side effects, e.g. burning/tingling sensations in the hands and feet, cold sensitivity and motor problems, *“I think neuropathy is a common enough side effect of many of the chemos so should be worth putting in there.”* (01-23).

An additional 20 concepts included in the conceptual model were not assessed by items in the EORTC QLQ C30/PAN26. Hair loss was identified as important but was not directly assessed although the impact of this may be indirectly captured in Item 48 “Have you felt physically less attractive as a result of your disease and treatment?”).Three concepts (change in stool colour, change in urine colour, surprise/shock at diagnosis) were specific to the pre-diagnosis/diagnosis patient experience and are perhaps likely less relevant to patients undergoing treatment. Sixteen other concepts were mentioned by ≤ 5 patients, suggesting that they may be less frequently occurring or of comparatively lesser importance to patients.

Conversely, Item 42 (Did you feel weak in your arms and legs?”) was not associated with any concept in the conceptual model. While patients had reported feeling weak, none discussed feeling weak in their arms and legs in particular. However, patients found this item to be relevant when directly questioned about it during cognitive debriefing.

### Interpretation and understanding

Overall most items were interpreted consistently by patients. However, some issues were apparent regarding item wording (Table 5).

**Table 5:** Interpretation and understanding of EORTC measures

Item	Difficulty
2. Do you have any trouble taking a long walk? 3. Do you have any trouble taking a short walk outside the house?	Patient interpretation of the distance that constituted ‘long’ and ‘short’ walk varied considerably
46. Did you have frequent bowel movements?	Interpretations of ‘frequently’ varied between patients; some considering ‘frequent bowel movements’ to mean ‘normal (or regular) bowel movements,’; others considered frequent bowel movements to indicate a problem (e.g. diarrhoea)
48. Have you felt physically less attractive as a result of your disease and treatment? 49. Have you been dissatisfied with your body?	Many patients responded to item 49 with reference to their physical appearance, potentially making this item redundant to item 48, ‘Have you felt physically less attractive as a result of your disease and treatment?’

### Response scale and options

With the exception of items 29 and 30, all items are scored on a 4-point Likert scale, (1 = ‘Not at all’ to 4 = ‘Very much’). Overall, patients found the response options understandable and appropriate. One patient suggested that a ‘middle-ground’ option would be beneficial for some questions because there was too great a difference between the second and third options of ‘a little bit’ and ‘quite a bit.’ Items 29 and 30 are scored on a 7-point numerical rating scale (NRS) where 1 indicates ‘very poor’ and 7 indicates ‘excellent’. This scale was used appropriately by all participants.

### Recall period

Overall, patients understood and used the recall period (“during the past week…”) where it was included. However, the first five items of the EORTC QLQ-C30/PAN26 include no clear recall period and, subsequently, the recall period used appeared to vary between patients.

## Discussion

This study triangulated data from multiple sources including published literature and qualitative interviews with clinical experts and patients with Stage III/IV pancreatic cancer. To our knowledge, this is the first qualitative study to explore patients’ experiences of metastatic pancreatic cancer and its treatments, identify relevant patient-centred outcomes for assessment in clinical trials and evaluate the conceptual relevance and acceptability of selected PRO instruments, in line with industry standards [9, 11]. Although the FACT-Hep is a potentially useful alternative PRO tool for use in pancreatic cancer, the EORTC QLQ-C30/PAN26 alone was evaluated in this study in order to minimise patient burden.

Findings from this research led to the development of a disease conceptual model; to our knowledge, this is the first conceptual model to provide a comprehensive representation of advanced Stage III/IV pancreatic cancer and its treatment. Pain was identified as particularly important to patients living with pancreatic cancer; pain was experienced frequently and affected many aspects of daily life. Although pain was not a consistent for all patients and could be managed; when it did occur, patients reported difficulty sleeping, eating, being unable to travel, drive, and take part in physical activities and hobbies.

Other frequent and bothersome important symptoms/side effects included significant tiredness, exhaustion and lack of energy. It was difficult to qualitatively distinguish whether this was a progressive symptom of disease and/or a side effect of treatments. The impact of tiredness/fatigue-related symptoms was considerable; patients were less able to complete daily activities, work and perform hobbies. They also reported impaired physical functioning and had to spend more time in bed or resting as a consequence. In addition, neuropathic treatment-related side effects were common in patients on chemotherapy.

Cognitive debriefing of the EORTC QLQ-C30/PAN26 allowed evaluation of content validity using methodology consistent with the FDA PRO Guidance [9]. No published studies were identified for comparison of these findings. This content validity evaluation identified strong coverage of pain, fatigue, gastrointestinal problems and dietary changes that were identified as important to patients. Overall, the findings of this study indicate that the EORTC QLQ-C30 pain scale and PAN26 pancreatic pain scale are both particularly salient to measure in this population. The EORTC QLQ-C30 fatigue domain may also offer an important score to understand patient performance status.

A key criticism of the EORTC QLQ-C30/PAN26, however, is the lack of any items to assess side effect symptom concepts related to chemotherapy-induced neuropathy. A review of available sources including the QLQ-C30 scoring manual and QLQ-C30 and PAN-26 development papers suggested that these concepts were not originally included and subsequently deleted for any reason (e.g. based on conceptual relevance or psychometric performance). This is unsurprising given that the issues and concepts raised as missing for this patient group on current treatments appear to be related to effects of chemotherapy and immunotherapy. As the PAN26 was developed in 1999, before platinums (folfirinox) and nab-paclitaxel were commonly used for pancreatic cancer, the absence of neuropathy items is understandable. However, to reflect the current treatment pathway and ensure the EORTC QLQ-C30/PAN-26 adequately captures the full patient experience of pancreatic cancer, it is advisable to include a question related to neuropathic symptoms. Such items may be identified from the EORTC item library (http://www.eortc.be/itemlibrary/). Alternatively, researchers could consider use of an additional patient-reported assessment of neuropathy alongside the EORTC QLQ-C3-/PAN26 to improve comprehensive measurement of patient experience.

Beyond conceptual coverage, findings suggested good usability as most items were interpreted consistently across the sample and completed without difficulty. Some items may benefit from altered wording to facilitate more consistent interpretation. The addition of a clear recall period to the first five items of the EORTC QLQ-C30 may improve standardisation of patient responses.

The psychometric performance of the EORTC QLQ-C30/PAN26 has previously been assessed in a locally advanced/metastatic pancreatic population. Evidence of internal consistency reliability [22] and criterion validity [23] was reported for the EORTC QLQ-C30; however, no evidence of additional psychometric assessment in a pancreatic Stage III/IV population was found. With the exception of evidence for internal consistency reliability in pancreatic cancer [24–26], gaps remain in our understanding of psychometric performance including test–retest reliability, convergent validity, known-groups validity and ability to detect change. Additionally, if extensive content changes are made, e.g. addition of items, the psychometric properties of the measure should be re-assessed.

Limitations of this study are acknowledged. The understanding of impacts and side effects was limited to the treatment types experienced by the interviewed sample of patients. Consequently, there was limited opportunity to understand the experiences associated with immunotherapy as only one participant had received immunotherapy; understandable given that it is not currently an approved therapy for patients with pancreatic cancer. The conceptual model could be further expanded with the emergence of new therapies and refined for different stages of disease. Furthermore, this was a single site study and therefore could have resulted in a sample with unrepresentative demographics, healthcare experiences and knowledge levels. However, this was somewhat mitigated as the study included patients who had travelled to the site from throughout the USA and so had experienced different care previously. Furthermore, data obtained from clinician interviews and published literature provided additional context.

## Conclusions

This qualitative interview study has provided an in-depth understanding of the ways in which pancreatic cancer and its treatment affect patients’ lives. The findings of this study support the conceptual relevance of the EORTC QLQ-C30 and PAN26 in a Stage III/IV pancreatic cancer population, although the absence of items to assess neuropathic treatment-related side effects should be considered by researchers and clinicians wishing to comprehensively assess impact on the patient experience. The EORTC QLQ-C30/PAN26 was well understood and completed with ease by the majority of patients, although minor revisions to item wording and addition of a clear recall period to items 1-5 could further improve usability.

Overall, in their current formats our study suggests that the EORTC QLQ-C30 and PAN26 provide a clear assessment of many of the concerns of patients living with locally advanced or metastatic pancreatic cancer.

## Electronic supplementary material

Below is the link to the electronic supplementary material.

Supplementary material 1 (DOCX 15 kb)

Supplementary material 2 (DOCX 15 kb)

Supplementary material 3 (DOCX 12 kb)

Supplementary material 4 (DOCX 62 kb)

Supplementary material 5 (DOCX 15 kb)

Supplementary material 6 (DOCX 167 kb)

Supplementary material 7 (DOCX 13 kb)

Supplementary material 8 (DOCX 12 kb)

Supplementary material 9 (DOCX 12 kb)

Supplementary material 10 (DOCX 34 kb)

## Data Availability

The datasets used and/or analysed during the current study are available from the corresponding author on reasonable request.
