# Supplementary material for: Exploring the patient experience of locally advanced or metastatic pancreatic cancer to inform patient-reported outcomes assessment
Source: Qual Life Res. 2019 Jul 4;28(11):2929–39. doi: 10.1007/s11136-019-02233-6 (PMC6803577; doi:10.1007/s11136-019-02233-6)
Supplement: Supplementary file 2 — Supplementary material 2 (DOCX 15 kb) [file 11136_2019_2233_MOESM2_ESM.docx]

Appendix 2: Search string used in Embase 1980 to 2014 Week 27

| # | Disease terms |
| --- | --- |
| 1 | exp Pancreatic Neoplasms/ |
| 2 | (pancrea$ adj5 neoplas$).tw. |
| 3 | (pancrea$ adj5 carcin$).tw. |
| 4 | (pancrea$ adj5 cancer$).tw. |
| 5 | (pancrea$ adj5 tumo$).tw. |
| 6 | (pancrea$ adj5 metasta$).tw. |
| 7 | (pancrea$ adj5 malig$).tw. |
| 8 | or/1-7 [Pancreatic cancer terms] |
| 9 | Qualitative Research/ |
| 10 | Nursing Methodology Research/ |
| 11 | Questionnaires/ |
| 12 | exp Attitude/ |
| 13 | Focus Groups/ |
| 14 | discourse analysis.mp. |
| 15 | content analysis.mp. |
| 16 | ethnographic research.mp. |
| 17 | ethnological research.mp. |
| 18 | ethnonursing research.mp. |
| 19 | constant comparative method.mp. |
| 20 | qualitative validity.mp. |
| 21 | purposive sample.mp. |
| 22 | observational method$.mp. |
| 23 | field stud$.mp. |
| 24 | theoretical sampl$.mp. |
| 25 | phenomenology/ |
| 26 | phenomenological research.mp. |
| 27 | life experience$.mp. |
| 28 | cluster sampl$.mp. |
| 29 | ethnonursing.af. |
| 30 | ethnograph$.mp. |
| 31 | phenomenol$.af. |
| 32 | grounded theory.mp. |
| 33 | (grounded adj (theor$ or study or studies or research or analys?s)).af. |
| 34 | (emic or etic or hermeneutic$ or heuristic$ or semiotic$).af. or (data adj1 saturat$).tw. or participant observ$.tw. |
| 35 | (action research or cooperative inquir$ or co operative inquir$ or co-operative inquir$).mp. |
| 36 | (humanistic or existential or experiential or paradigm$).mp. |
| 37 | human science.tw. |
| 38 | biographical method.tw. |
| 39 | qualitative validity.af. |
| 40 | purposive sampl$.af. |
| 41 | theoretical sampl$.af. |
| 42 | ((purpos$ adj4 sampl$) or (focus adj group$)).af. |
| 43 | (life world or life-world or conversation analys?s or personal experience$ or theoretical saturation).mp. |
| 44 | lived experience$.tw. |
| 45 | life experience$.mp. |
| 46 | cluster sampl$.mp. |
| 47 | (theme$ or thematic).mp. |
| 48 | categor$.mp. |
| 49 | observational method$.af. |
| 50 | field stud$.mp. |
| 51 | focus group$.af. |
| 52 | questionnaire$.mp. |
| 53 | content analysis.af. |
| 54 | thematic analysis.af. |
| 55 | constant comparative.af. |
| 56 | discourse analys?s.af. |
| 57 | ((discourse$ or discurs$) adj3 analys?s).tw. |
| 58 | (constant adj (comparative or comparison)).af. |
| 59 | narrative analys?s.af. |
| 60 | heidegger$.tw. |
| 61 | colaizzi$.tw. |
| 62 | speigelberg$.tw. |
| 63 | (van adj manen$).tw. |
| 64 | (van adj kaam$).tw. |
| 65 | (merleau adj ponty$).tw. |
| 66 | husserl$.tw. |
| 67 | giorgi$.tw. |
| 68 | foucault$.tw. |
| 69 | (corbin$ adj2 strauss$).tw. |
| 70 | (strauss$ adj2 corbin$).tw. |
| 71 | (glaser$ adj2 strauss$).tw. |
| 72 | glaser$.tw. |
| 73 | findings.af. |
| 74 | interview$.af. or Interviews/ |
| 75 | qualitative.af. |
| 76 | or/9-75 [Qualitative terms] |
| 77 | 8 and 76 |
| 78 | limit 77 to yr="2000 -Current" |
| 79 | limit 78 to humans |
| 80 | limit 79 to adult <18 to 64 years> |
| 81 | limit 80 to english language |
