# Supplementary material for: Exploring the patient experience of locally advanced or metastatic pancreatic cancer to inform patient-reported outcomes assessment
Source: Qual Life Res. 2019 Jul 4;28(11):2929–39. doi: 10.1007/s11136-019-02233-6 (PMC6803577; doi:10.1007/s11136-019-02233-6)
Supplement: Supplementary file 3 — Supplementary material 3 (DOCX 12 kb) [file 11136_2019_2233_MOESM3_ESM.docx]

Appendix 3: Search string used in PsycINFO

| # | Search term |
| --- | --- |
| 1 | Qualitative |
| 2 | Pancreatic cancer |
| 3 | Pancreatic neoplasm |
| 4 | Pancreatic carcinoma |
| 5 | Pancreatic tumour |
| 6 | Pancreatic metastatic |
| 7 | Pancreatic malignant |
| 8 | Or/2-7 |
| 9 | 1 and 8 |
