# Supplementary figures and images for: Exploring the patient experience of locally advanced or metastatic pancreatic cancer to inform patient-reported outcomes assessment
Source: Qual Life Res. 2019 Jul 4;28(11):2929–39. doi: 10.1007/s11136-019-02233-6 (PMC6803577; doi:10.1007/s11136-019-02233-6)

Appendix 4: PRISMA diagram for qualitative literature review


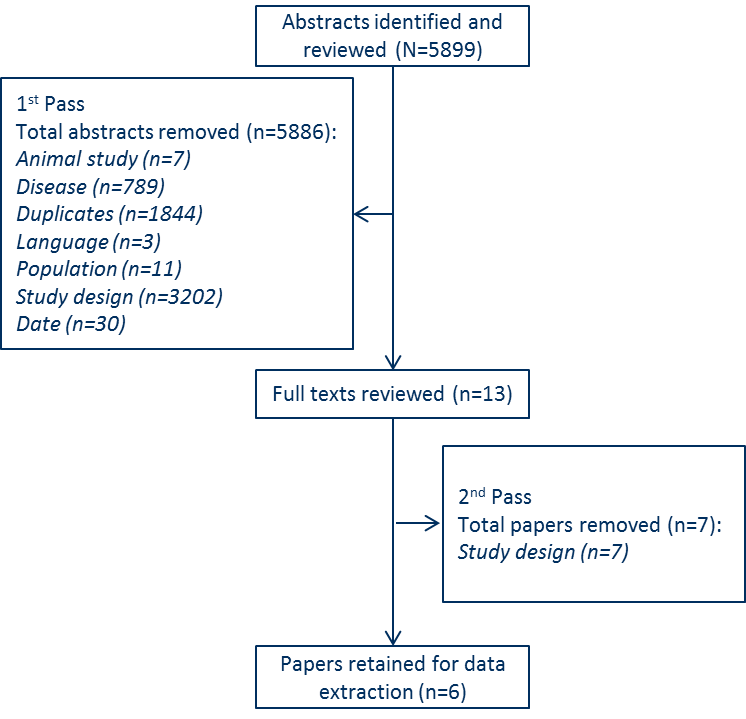

Supplement: Supplementary file 4 — Supplementary material 4 (DOCX 62 kb) [file 11136_2019_2233_MOESM4_ESM.docx]
