# Supplementary material for: Exploring the patient experience of locally advanced or metastatic pancreatic cancer to inform patient-reported outcomes assessment
Source: Qual Life Res. 2019 Jul 4;28(11):2929–39. doi: 10.1007/s11136-019-02233-6 (PMC6803577; doi:10.1007/s11136-019-02233-6)
Supplement: Supplementary file 5 — Supplementary material 5 (DOCX 15 kb) [file 11136_2019_2233_MOESM5_ESM.docx]

Appendix 5: Inclusion and exclusion criteria for qualitative literature review

| Criteria | Include | Exclude |
| --- | --- | --- |
| Disease | - Pancreatic cancer | - Diseases other than pancreatic cancer |
| Study design | - Any study designs which report qualitative patient experiences (including interviews, focus groups [e.g., grounded theory, ethnography etc.] and survey designs) | - Non-qualitative studies |
| Population | - Adults (≥18 years) - Human studies | - Patients <18 years of age - Animal studies - Non-patient population (e.g. caregiver) |
| Publication year | - 2000 to 2014 | - Published prior to 2000 |
| Publication language | - English language | - Non English-language (Abstracts of highly relevant non-English language studies will be flagged for discussion regarding potential translation) |
