# Supplementary material for: Exploring the patient experience of locally advanced or metastatic pancreatic cancer to inform patient-reported outcomes assessment
Source: Qual Life Res. 2019 Jul 4;28(11):2929–39. doi: 10.1007/s11136-019-02233-6 (PMC6803577; doi:10.1007/s11136-019-02233-6)
Supplement: Supplementary file 6 — Supplementary material 6 (DOCX 167 kb) [file 11136_2019_2233_MOESM6_ESM.docx]

**Appendix 6:** Qualitative patient interviews saturation grid: conceptual saturation analysis

| **Conceptual model item** | **Patients, in chronological order of date of interview** | | | | | | | | | | | | | | | | | | | | | | | |
| --- | --- | --- | --- | --- | --- | --- | --- | --- | --- | --- | --- | --- | --- | --- | --- | --- | --- | --- | --- | --- | --- | --- | --- | --- |
|  | **Set 1** | | | | | | | | **Set 2** | | | | | | | | **Set 3** | | | | | | | |
|  | **01-01** | **01-02** | **01-03** | **01-04** | **01-07** | **01-06** | **01-08** | **01-09** | **01-12-** | **01-16** | **01-15** | **01-17** | **01-18** | **01-20** | **01-05** | **01-19** | **02-25** | **01-24** | **01-11** | **02-26** | **01-21** | **01-23** | **02-27** | **02-29** |
| Pain/ ache | X | X | X | X | X | X | X | X | X | X | X |  | X | X | X | X | X | X | X |  | X | X |  | X |
| Fear/ worry | X | X | X | X |  | X | X | X | X |  | X |  | X |  |  |  | X |  |  |  | X | X |  | X |
| Tired/ exhausted | X | X |  | X | X | X | X | X | X | X | X | X | X | X | X | X |  | X | X | X | X | X | X |  |
| Surprise/ shock (at diagnosis) | X |  | X | X | X | X |  |  | X |  |  |  | X | X | X | X | X |  |  |  |  | X |  |  |
| Diarrhoea | X |  | X | X | X |  |  |  |  | X | X | X | X | X | X | X |  |  | X | X |  | X | X |  |
| Difficulty sleeping through the night | X |  | X |  | X | X |  |  |  |  |  | X | X |  |  | X |  | X |  |  |  |  | X | X |
| Yellow skin/ eyes | X |  | X |  |  |  |  |  | X |  |  | X | X |  | X |  | X |  | X |  |  |  |  |  |
| Pruritus/ itching | X |  | X |  |  |  |  |  |  | X |  |  | X |  | X |  |  |  |  |  |  |  |  |  |
| Change in stool colour | X |  | X |  |  |  |  |  |  |  |  |  |  |  |  |  |  |  |  |  |  |  |  |  |
| Sad/ upset/ depressed | X |  |  | X | X | X | X | X | X |  |  |  | X |  |  |  |  | X |  |  |  | X |  |  |
| Gas/ Bloating | X |  |  | X | X |  |  | X |  | X |  |  |  | X |  |  | X | X |  |  |  | X |  | X |
| Indigestion | X |  |  | X |  |  |  |  |  |  |  |  | X |  |  |  |  |  |  | X |  |  |  |  |
| Lack of energy | X |  |  |  | X |  |  | X |  |  |  | X | X |  | X | X |  | X | X |  | X | X | X | X |
| Change in urine colour | X |  |  |  |  |  |  |  | X |  |  | X |  |  | X |  | X |  |  |  |  |  | X |  |
| Weight loss |  | X | X | X | X | X |  | X | X | X |  |  | X | X | X |  |  | X | X |  | X | X | X | X |
| Feeling overwhelmed |  | X | X |  |  |  |  |  |  |  |  |  |  |  | X | X |  | X |  |  |  |  |  |  |
| Increased resting/ napping |  | X |  |  | X | X | X | X | X | X | X | X | X | X |  |  |  | X | X | X | X |  | X | X |
| Hair loss |  | X |  |  | X |  | X |  |  |  | X |  | X | X |  | X |  | X | X |  |  | X | X |  |
| Nausea |  | X |  |  |  |  | X |  | X |  | X | X | X | X |  | X | X | X |  | X | X | X |  | X |
| Change in diet |  |  | X | X | X | X |  | X |  |  |  | X |  | X |  | X | X | X |  |  |  |  |  |  |
| Eating less/ lack of appetite |  |  | X |  | X | X | X | X | X |  | X |  | X | X | X |  | X | X | X | X | X | X | X | X |
| Unable/ harder to do chores |  |  | X |  | X |  |  |  |  | X |  |  |  | X |  |  | X |  |  | X |  |  | X |  |
| Unable to work full time |  |  | X |  |  | X |  |  |  |  |  |  |  |  |  |  | X | X |  |  |  | X | X |  |
| Difficulty walking |  |  | X |  |  |  |  | X |  |  | X |  | X | X | X |  |  |  |  |  |  |  | X |  |
| Vomiting |  |  | X |  |  |  |  |  | X |  |  |  | X |  |  | X | X |  | X | X | X |  |  | X |
| Sensitivity to cold |  |  |  | X |  | X |  |  |  |  |  | X | X | X | X | X | X | X |  |  |  | X |  |  |
| Change in taste |  |  |  | X |  |  | X |  |  | X | X |  |  |  | X |  | X |  | X |  |  | X | X |  |
| Constipation |  |  |  | X |  |  |  |  | X |  |  |  |  |  |  | X | X |  |  |  |  | X |  |  |
| Annoyance/ frustration |  |  |  | X |  |  |  |  | X |  |  |  |  |  |  |  |  |  |  |  |  |  |  |  |
| Dizziness |  |  |  | X |  |  |  |  |  |  |  |  | X |  |  |  | X |  | X |  |  |  |  |  |
| Vision symptoms |  |  |  | X |  |  |  |  |  |  |  |  |  |  |  |  |  |  |  |  |  | X |  |  |
| Financial burden |  |  |  | X |  |  |  |  |  |  |  |  |  |  |  |  |  |  |  |  |  |  |  |  |
| Unable to drive |  |  |  |  | X | X |  |  |  |  | X |  |  |  |  |  | X |  |  |  |  | X |  |  |
| Poor concentration |  |  |  |  | X | X |  |  |  |  |  |  |  |  |  |  |  |  |  |  |  | X |  |  |
| Dehydration |  |  |  |  | X |  |  |  |  |  |  |  | X | X | X |  |  |  |  |  |  |  | X |  |
| Reduced social life |  |  |  |  | X |  |  |  |  |  |  |  |  | X |  |  |  | X |  | X |  |  | X |  |
| Dry skin at radiation site |  |  |  |  | X |  |  |  |  |  |  |  |  |  |  |  |  |  |  |  |  |  |  |  |
| Poor memory |  |  |  |  |  | X |  |  |  |  | X |  |  | X |  |  |  |  |  |  |  | X |  |  |
| Numbness/ burning/ tingling in hands/ feet |  |  |  |  |  | X |  |  |  |  |  |  | X |  |  | X | X |  |  |  | X | X |  |  |
| Poor motor control |  |  |  |  |  | X |  |  |  |  |  |  |  | X |  |  |  |  |  |  |  | X | X |  |
| Unable to take part in hobbies |  |  |  |  |  |  | X |  |  |  |  | X |  |  |  |  |  | X | X |  | X |  |  | X |
| Shortness of breath |  |  |  |  |  |  | X |  |  |  |  |  |  |  |  |  |  |  | X |  |  |  |  | X |
| Swollen legs, cellulitis |  |  |  |  |  |  | X |  |  |  |  |  |  |  |  |  |  |  |  |  |  |  |  |  |
| Fever |  |  |  |  |  |  |  | X | X |  |  |  |  |  |  |  |  |  |  |  |  |  |  |  |
| Dry mouth |  |  |  |  |  |  |  |  | X |  | X |  |  |  |  |  |  |  |  |  |  |  |  |  |
| Swollen skin at radiation site |  |  |  |  |  |  |  |  | X |  |  |  |  |  |  |  |  |  |  |  |  |  |  |  |
| Feeling unattractive |  |  |  |  |  |  |  |  |  |  |  |  | X |  |  |  |  |  | X |  |  |  |  | X |
| Drooling |  |  |  |  |  |  |  |  |  |  |  |  |  |  |  | X |  |  |  |  |  |  |  |  |
| Weak urinary stream |  |  |  |  |  |  |  |  |  |  |  |  |  |  |  | X |  |  |  |  |  |  |  |  |
| Reduced sex life |  |  |  |  |  |  |  |  |  |  |  |  |  |  |  | X |  |  |  |  |  |  |  |  |
| Unable to travel |  |  |  |  |  |  |  |  |  |  |  |  |  |  |  |  | X | X |  |  |  |  | X |  |
| Speech, communication difficulties |  |  |  |  |  |  |  |  |  |  |  |  |  |  |  |  | X |  |  |  |  |  |  | X |
| Frequent urination |  |  |  |  |  |  |  |  |  |  |  |  |  |  |  |  | X |  |  |  |  |  |  |  |
| Hiccups |  |  |  |  |  |  |  |  |  |  |  |  |  |  |  |  |  | X |  | X |  |  |  |  |
| Blood clots |  |  |  |  |  |  |  |  |  |  |  |  |  |  |  |  |  | X |  |  |  |  |  |  |
| Burns at radiation site^†^ |  |  |  |  |  |  |  |  |  |  |  |  |  |  |  |  |  |  |  |  |  |  |  |  |
| Chills^†^ |  |  |  |  |  |  |  |  |  |  |  |  |  |  |  |  |  |  |  |  |  |  |  |  |

^†^Concepts mentioned by clinicians only
