# Supplementary material for: Exploring the patient experience of locally advanced or metastatic pancreatic cancer to inform patient-reported outcomes assessment
Source: Qual Life Res. 2019 Jul 4;28(11):2929–39. doi: 10.1007/s11136-019-02233-6 (PMC6803577; doi:10.1007/s11136-019-02233-6)
Supplement: Supplementary file 7 — Supplementary material 7 (DOCX 13 kb) [file 11136_2019_2233_MOESM7_ESM.docx]

Appendix 7: PRO literature review search string (Pubmed)

| # | Search term |
| --- | --- |
| 1 | Pancreatic cancer |
| 2 | Patient reported outcome |
| 3 | PRO |
| 4 | Questionnaire |
| 5 | Survey |
| 6 | or/2-6 |
| 7 | 1 and 6 |
