# Supplementary material for: Exploring the patient experience of locally advanced or metastatic pancreatic cancer to inform patient-reported outcomes assessment
Source: Qual Life Res. 2019 Jul 4;28(11):2929–39. doi: 10.1007/s11136-019-02233-6 (PMC6803577; doi:10.1007/s11136-019-02233-6)
Supplement: Supplementary file 8 — Supplementary material 8 (DOCX 12 kb) [file 11136_2019_2233_MOESM8_ESM.docx]

Appendix 8: Search terms used in PROQOLID

| # | Search term |
| --- | --- |
| 1 | Pathology=neoplasms |

The PROQOLID search function did not allow for a more specific search of pancreatic cancer alone.
