# Supplementary material for: Exploring the patient experience of locally advanced or metastatic pancreatic cancer to inform patient-reported outcomes assessment
Source: Qual Life Res. 2019 Jul 4;28(11):2929–39. doi: 10.1007/s11136-019-02233-6 (PMC6803577; doi:10.1007/s11136-019-02233-6)
Supplement: Supplementary file 10 — Supplementary material 10 (DOCX 34 kb) [file 11136_2019_2233_MOESM10_ESM.docx]

Appendix 10: PRO measures reported in at least two publications or identified as being pancreatic cancer-specific measures

| **Measure** | **Number of publications reporting PRO data** | **Specifically developed for pancreatic cancer patients?** |
| --- | --- | --- |
| European Organization for Research and Treatment of Cancer Quality of Life Questionnaire Core 30 (EORTC QLQ-C30) | 38 | No |
| European Organization for Research and Treatment of Cancer Quality of Life Questionnaire Pancreatic 26 (EORTC QLQ-PAN26) | 19 | Yes |
| Functional Assessment of Cancer Therapy Hepatobiliary (FACT-Hep) | 6 | Yes* |
| Pain Visual Analogue Scale (VAS) | 5 | No |
| Edmonton Symptom Assessment System (ESAS)Short Form Health Survey 36 (SF-36) | 4 | No |
| Functional Assessment of Cancer Therapy-General (FACT-G) | 3 | No |
| Brief Pain Inventory (BPI) | 2 | No |
| National Comprehensive Cancer Network / Functional Assessment of Cancer Therapy – Hepatobiliary Symptom Index (NCCN-FACT FHSI-18) | 2 | Yes* |
| Functional Assessment of Cancer Therapy – Hepatobiliary Symptom Index (FHSI-8) | 2 | Yes* |
| Hamilton Depression Rating Scale- 24 (HAMD-24) | 2 | No |
| Gastrointestinal Quality of Life Index (GIQLI) | 2 | No |
| Functional Assessment of Cancer Therapy – Pancreatic (FACT-PA) | 1 | Yes |
| Pancreatic Cancer Disease Impact Score (PACADI) | 1 | Yes |
| Memorial Pain Assessment Card (MPAC) | N/A^†^ | No |

Abbreviations: N/A, not applicable.

*Developed for patients with hepatobiliary cancer, which includes cancers of the pancreas, liver, gallbladder and bile duct amongst others.

^†^Measure identified via hand searching and not via PRO literature review.
